# Synthesis and antimicrobial evaluation of some novel 1,2,4-triazole and 1,3,4-thiadiazole derivatives

**DOI:** 10.1007/s00044-012-0302-9

**Published:** 2012-11-11

**Authors:** Łukasz Popiołek, Urszula Kosikowska, Liliana Mazur, Maria Dobosz, Anna Malm

**Affiliations:** 1 Department of Organic Chemistry, Faculty of Pharmacy, Medical University, Lublin, Poland; 2 Department of Pharmaceutical Microbiology, Faculty of Pharmacy, Medical University, Lublin, Poland; 3 Department of General and Coordination Chemistry, Faculty of Chemistry, Maria Curie-Sklodowska University, Lublin, Poland

**Keywords:** Antimicrobial activity, 1,2,4-Triazole derivatives, 1,3,4-Thiadiazole derivatives

## Abstract

**Abstract:**

This study presents the synthesis and spectral analysis of new derivatives of 1,2,4-triazole-3-thione and 1,3,4-thiadiazole. New compounds were prepared by cyclization reaction of acyl thiosemicarbazide derivatives in the presence of alkaline and acidic media. All synthesized compounds were screened for their in vitro antibacterial activity by using the agar dilution technique. Six of the compounds had potential activity against Gram-positive bacteria (minimal inhibitory concentration [MIC] = 15.63–500 μg/mL). Some compounds showed good activity especially against *Bacillus subtilis* ATCC 6633 (MIC = 15.63–250 μg/mL), *Staphylococcus aureus* ATCC 25923 (MIC = 31.25–250 μg/mL), and *Micrococcus luteus* ATCC 10240 (MIC = 125–250 μg/mL).

**Graphical abstract:**

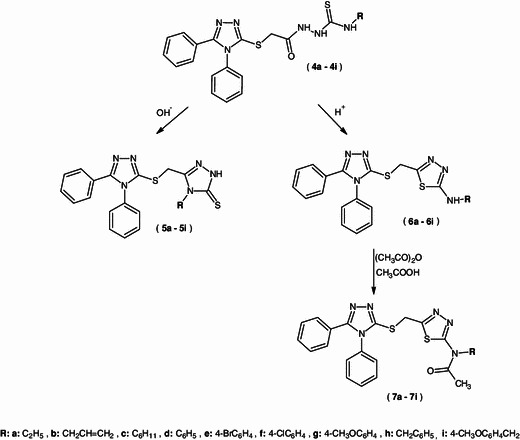

## Introduction

For the last few decades, there has been a tremendous growth of research in the synthesis of nitrogen and sulfur containing heterocyclic derivatives because of their utility in various applications, such as pharmaceuticals, propellants, explosives, and pyrotechnics.

The recent literature is enriched with progressive findings about the synthesis and pharmacological action of triazole and thiadiazole derivatives. Heterocycles bearing 1,2,4-triazole and 1,3,4-thiadiazole moiety are reported to show a broad spectrum of biologic activity such as analgesic (Turan-Zitouni *et al*., 1999), antiphlogistic (Harish *et al*., 2008; El Shehry *et al*., 2010; Schenone *et al*., 2006), anticonvulsant (Dogan *et al*., 2002; Almasirad *et al*., 2004), antitumor (Duran *et al*., 2002; Kumar *et al*., 2010), antiviral (Al-Soud *et al*., 2004), antifungal (Collin *et al*., 2003; Wei *et al*., 2006), antibacterial (Ulusoy *et al*., 2001; Gülerman *et al*., 2001; Padmavathi *et al*., 2009; Demirbas *et al*., 2009; Liesen *et al*., 2010), and antitubercular action (Klimešová *et al*., 2004; Gadad *et al*., 2004; Shiradkar *et al*., 2007). A large number of ring systems containing triazoles and thiadiazoles have been incorporated into a wide variety of therapeutically interesting drug candidates. Some of them are approved as drugs, for example, alprazolam (Pick, 1997), etizolam (Shiroki *et al*., 1976), or vibunazole (Holmwood *et al*., 1982). Vorozole, letrozole, and anastrozole are non-steroidal drugs used for the treatment of cancer (Clemons *et al*., 2004). Triazoles are also used as intermediates for the synthesis of antifungal agents such as fluconazole, voriconazole, and itraconazole (Bailey *et al*., 1990; McGinnis *et al*., 1997).

In continuation of our research program on the synthesis of 1,2,4-triazole and 1,3,4-thiadiazole compounds exhibiting biologic activity, it was thought to be interesting to synthesize new antimicrobial agents, especially when the development of resistance of pathogenic bacteria toward available antibiotics is rapidly becoming a major worldwide problem. The designing of new compounds to deal with resistant bacteria has become one of the most important areas of antibacterial research today. In addition, primary and opportunistic microbial infections continue to increase rapidly because of the increased number of immunocompromised patients.

Keeping in mind the above facts, we designed and synthesized series of some new 1,2,4-triazole-3-thione and 1,3,4-thiadiazole derivatives and evaluated their in vitro antibacterial activity.

## Results and discussion

### Chemistry

The substituted 1,2,4-triazole and 1,3,4-thiadiazole derivatives are generally obtained by the cyclization reaction of thiosemicarbazide derivatives, which is dependent not only on the pH of the medium, but also on the nature of substituents in thiosemicarbazide derivatives (Dobosz and Pachuta-Stec, 1995, 1996). The presence of alkaline media usually promotes the reaction of cyclization to obtain 1,2,4-triazole systems, whereas in acidic media, 1,3,4-thiadiazole derivatives were obtained.

4,5-Diphenyl-4*H*-1,2,4-triazole-3-thione **1** was a starting material for the synthesis of new compounds, which consist of two 1,2,4-triazole systems or 1,2,4-triazole and 1,3,4-thiadiazole systems connected with the S-methylene group. Compound **1** was obtained by the cyclization reaction of 1,4-diphenyl thiosemicarbazide in alkaline media. In the next step, compound **1**, which can exist in two tautomeric forms, was submitted to the reaction with ethyl bromoacetate in the presence of sodium ethanolate. The reaction let us obtain ethyl 2-[(4,5-diphenyl-4*H*-1,2,4-triazol-3-yl)sulfanyl] acetate (**2**). The direction of this reaction to form a thio derivative of compound **1** was revealed and confirmed by X-ray crystallography (Dobosz *et al*., 1996). The mechanism of this reaction as a nucleophilic substitution on the sulfur atom had been studied and investigated earlier (Wujec and Paneth, 2007).

Subsequently, compound **2** was converted to hydrazide **3** in reaction with 100 % hydrazine hydrate. Then, reactions of hydrazide **3** with various isothiocyanates were performed in two ways.

All new thiosemicarbazide derivatives **4a**–**l** were obtained by heating reactants in an oil bath; temperatures were selected experimentally (*t* = 50–110 °C). Thiosemicarbazide derivatives **4a**, **c**, **d** were products of the reaction of hydrazide **3** with appropriate isothiocyanates in the presence of diethyl ether carried in room temperature.

A new group of compounds, which consist of two 1,2,4-triazole-3-thione derivatives **5a**–**i**, were acquired in cyclization reaction with 2 % aqueous solution of sodium hydroxide of new acyl thiosemicarbazide derivatives **4a**–**i**.

In three cases, the cyclization reaction of thiosemicarbazide derivatives **4j**–**l** in alkaline media was accompanied by hydrolysis. The [(4,5-diphenyl-4*H*-1,2,4-triazol-3-yl)sulfanyl] acetic acid **8** was obtained in cyclization of 4-ethoxycarbonyl-1-substituted thiosemicarbazide **4j**. This compound was described earlier, but it was obtained in a different way (Kaplaushenko *et al*., 2008). The cyclization in alkaline media of the thiosemicarbazide which contains the ethoxycarbonylmethyl group **4k** and benzoyl **4l** in the fourth position led us to obtain substituted 1,2,4-triazole-3-thione derivatives **9**, **10**. These compounds were subjected to the reaction with pyrrolidine and formaldehyde to get new *N*-substituted 1,2,4-triazole-3-thione derivatives **11**, **12**.

The thiosemicarbazide derivatives **4a**–**i** were also submitted to the cyclization reaction in acidic media. In this way, we were able to obtain new compounds which consist of 1,2,4-triazole-3-thione and 1,3,4-thiadiazole system, that is (5-aminosubstituted)-2-{[(4,5-diphenyl-4*H*-1,2,4-triazol-3-yl)sulfanyl]methyl}-1,3,4-thiadiazole **6a**–**i**. Afterward, the derivatives of *N*,*N*-disubstituted acetamide **7a**–**i** were obtained by the acylation reaction of 2,5-disubstituted-1,3,4-thiadiazoles **6a**–**i** with acetic anhydride.

The mechanism of cyclization of thiosemicarbazide was investigated earlier (Siwek and Paneth, 2007). It was proved that the direction of cyclization is dependent on the nature of substituents and acidic or alkaline media (Siwek *et al*., 2010).

**Scheme 1:**
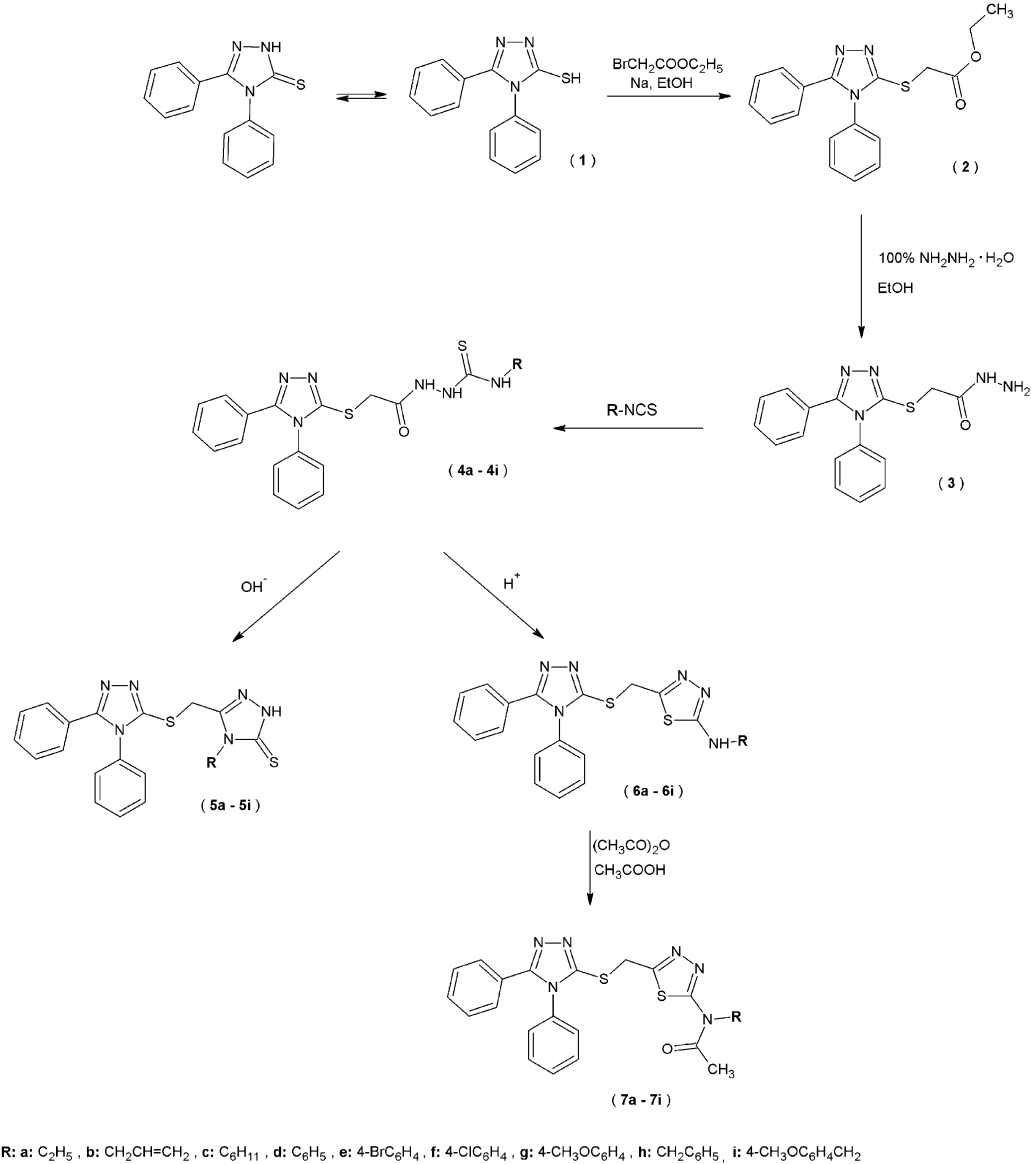
Synthesis of new derivatives of thiosemicabrazide, 1,2,4-triazole-3-thione and 1,3,4-thiadiazole

**Scheme 2:**
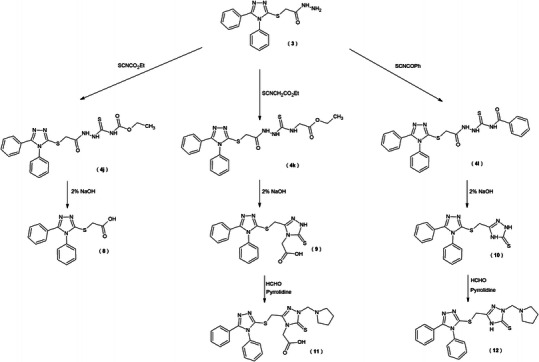
Synthesis of new derivatives of 1,2,4-triazole-3-thione

The structure of all obtained compounds was confirmed by elementary analysis, IR and ^1^H NMR spectra. Some of the compounds were also submitted to ^13^C NMR and MS spectra analyses. The crystal structure of the representative compound **2** was determined by the single-crystal X-ray analysis. The reactions were performed according to Schemes 1 and 2.

In the IR spectra of the thiosemicarbazide derivatives **4a**–**l**, the following characteristic absorption bands were observed: about 1,700 cm^−1^ corresponding to the C=O group and in the range of 1,300 cm^−1^ corresponding to the C=S group. Compounds which consist of two 1,2,4-triazole systems **5a**–**i**, **9**, **10** had absorption bands: about 1,300 cm^−1^ (C=S group), about 1,500 cm^−1^ (C–N group), in the range of 1,600 cm^−1^ (C=N group), and about 3,100–3,200 cm^−1^ (NH group). Then, in the IR spectra of the new derivatives of 1,3,4-thiadiazole **6a**–**i**, the following characteristic absorption bands were observed: in the range of 1,500 cm^−1^ corresponding to the C–N group and in the range of 1,600 cm^−1^ corresponding to the C=N group and about 3,200 cm^−1^ for the NH group. Compounds **7a**–**i**, **11** had a characteristic absorption band at about 1,700 cm^−1^ for the C=O group.

^1^H NMR spectra of the thiosemicarbazide derivatives **4a**–**l** show three proton signals typical for the NH group in the δ 8.32–12.87 ppm range, whereas for the new compounds consisting of two 1,2,4-triazole system **5a**–**i**, **9**, **10,** one proton signal of the NH group was observed in the δ 13.62–14.13 ppm range. The 1,3,4-thiadiazole derivatives **6a**–**i** had one typical proton signal of the NH group in the δ 9.35–10.47 ppm range. Derivatives of *N*,*N*-disubstituted acetamide **7a**–**i** had one proton signal of the CH_3_ group in the δ 2.06–2.16 ppm range. Compound **11** had one proton signal for the OH group (δ 13.68 ppm) and for the pyrrolidine substituent. Similarly, 4,5-disubstituted-2-(pyrrolidin-1-ylmethyl)-1,2,4-triazole-3-thione **12** had one typical proton signal for the NH group (δ 14.68 ppm) and for the pyrrolidine substituent.

**Fig. 1:**
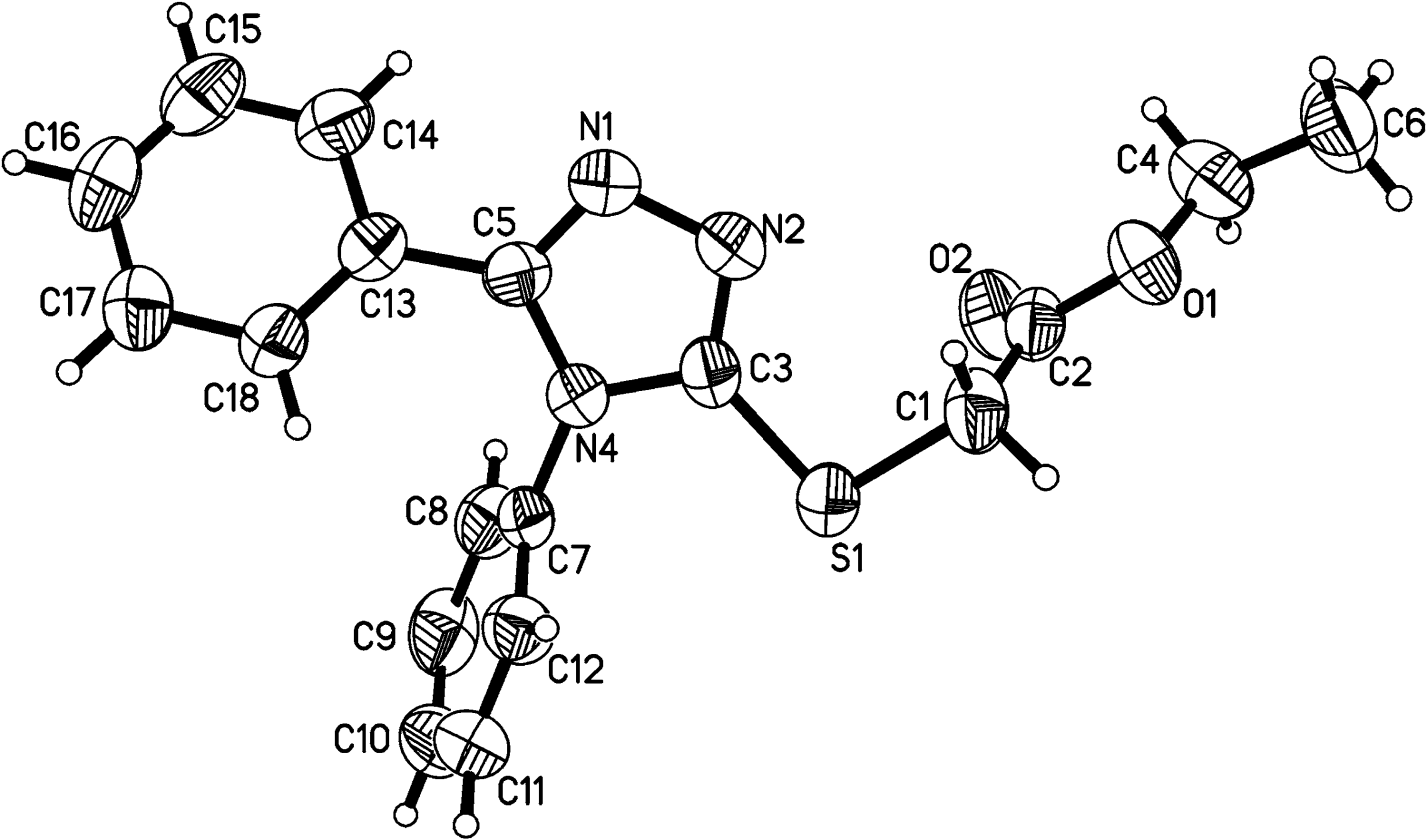
Molecular structure of **2** with atom-labeling scheme. Displacement ellipsoids are drawn at the 50 % probability level. Selected bond distances (Ǻ): C3–S1 1.738(2), C1–S1 1.789(3), C1–C2 1.494(4), C2–O1 1.321(3), C2–O2 1.191(3), C4–O1 1.460(3)

Compound **2** crystallizes in the monoclinic space group *P*2_1_/*n* with one molecule in the asymmetric unit of the crystal. The diffraction study confirmed that the molecule contained the 1,2,4-triazole ring, substituted at C3, N4, and C5 atoms by thioacetate moiety and two phenyl rings, respectively (Fig. 1). The chain of atoms from S1 to ethyl C4 is almost planar (rmsd = 0.006 Å); a higher twist (4.56°) is observed around the C4–O1 bond in the solid state. The best plane of the atoms of thioacetate unit intersects that of the 1,2,4-triazole ring at the angle of 81.4(1)°. The carbonyl C2=O2 group in **2** is *cis* oriented with respect to the thioether S1 atom. What is more, it seems to be preferred in thioacetate derivatives in the solid state (CSD, V.5.33, Allen, 2002). The geometric parameters of the ester group are within normal ranges (International Tables for Crystallography, 1995). Likewise, the S1–C3 and S1–C1 distances, being of 1.738(2) and 1.789(3) Å, are in agreement with the single thioether C–S bonds. The most characteristic feature of the crystal of **2** is the presence of centrosymmetric molecular dimers. The “head-to-head” oriented molecules within the dimer form short S1···O2^i^ [3.268(3) Å; (i) 1 − *x*, −*y*, −*z*] contacts which might be attractive in their nature (Ramasubbu and Parthasarathy, 1989).

### Microbiology

On the basis of the preliminary results obtained by the agar dilution method, it was shown that some of the newly synthesized compounds had the potential activity against reference strains of Gram-positive bacteria. None of the compounds had inhibitory effect on the Gram-negative bacteria growth.

According to Table 1, on the basis of minimal inhibitory concentration (MIC) values obtained by the broth microdilution method, it was shown that the highest activity had compound **4l** with MIC = 31.25 μg/mL against *Staphylococcus aureus* ATCC 25923, MIC = 125 μg/mL against *Staphylococcus epidermidis* ATCC 12228, *Bacillus cereus* ATCC 10876, and *Micrococcus luteus* ATCC 10240 or MIC = 250 μg/mL against *S. aureus* ATCC 6538 and *Bacillus subtilis* ATCC 6633. Compound **6h** was also active especially against *B. subtilis* ATCC 6633 with MIC = 15.63 μg/mL and with MIC = 125 μg/mL against *M. luteus* ATCC 10240 or MIC = 250 μg/mL against *S. aureus* ATCC 25923.

**Table 1:** The inhibitory activities of newly synthesized compounds against Gram-positive bacteria on the basis of MIC (μg/mL) values determined by broth microdilution method

Species:	Sa25923	Sa6538	Se12228	Bs6633	Bc10876	Ml10240
Compounds	MIC (μg/mL)					
**4c**	>1000	>1000	>1000	**1000**	**1000**	**1000**
**4i**	**500**	**1000**	**500**	**500**	**1000**	**1000**
**4j**	>1000	>1000	**1000**	**500**	**500**	**1000**
**4l**	**31.25**	**250**	**125**	**250**	**125**	**125**
**5b**	**1000**	**1000**	**1000**	**1000**	**1000**	**1000**
**5c**	**500**	**250**	**500**	**500**	**1000**	**250**
**5d**	**1000**	>1000	>1000	>1000	**500**	>1000
**5g**	**1000**	>1000	>1000	>1000	**500**	>1000
**5h**	**1000**	**1000**	**1000**	>1000	>1000	1000
**5i**	>1000	**1000**	>1000	>1000	>1000	>1000
**6h**	**250**	nd	**500**	**15.63**	nd	**125**
Cefuroxime	0.49	1.95	0.24	0.49	62.5	0.49

Bold values indicate the lowest MIC *nd* Not determined, *Sa25923* *S. aureus* ATCC 25923, *Sa6538* *S. aureus* ATCC 6538, *Se12228* *S. epidermidis* ATCC 12228, *Bs6633* *B. subtilis* ATCC 6633, *Bc10876* *B. cereus* ATCC 10876, *Ml10240* *M. luteus* ATCC 10240

The somewhat lower activity against reference strains of Gram-positive bacteria was shown by compound **5c** (MIC values from 250 to 1,000 μg/mL). According to our results, MICs of cefuroxime, which has been extensively used to treat bacterial infections, were 0.24–1.95 μg/mL for *Staphylococcus* species and 0.49–62.5 μg/mL for the other Gram-positive bacteria.

With our research, it has been established that the introduction of the benzoyl group in thiosemicarbazide and the benzyl group in 1,3,4-thiadiazole derivative yielded active compounds endowed with a wide spectrum of antimicrobial activities.

The compounds **4l** and **6h** with potential activity against the reference strains of Gram-positive bacteria may be regarded as precursor compounds for searching for new derivatives showing antimicrobial activity against pathogenic (e.g. *S. aureus*) or opportunistic (e.g. *S. epidermidis*, *M. luteus*, *B. subtilis*, or *B. cereus*) bacteria.

## Experimental

### Chemistry

Melting points were determined in Fisher–Johns blocks (Pittsburgh, US) and presented without any corrections. The IR spectra (*ν*, cm^−1^) were recorded in KBr tablets using a Specord IR-75 spectrophotometer (Germany). The NMR spectra were recorded on a Bruker Avance 300 apparatus (Bruker BioSpin GmbH, Rheinstetten/Karlsruhe, Germany) in dimethyl sulfoxide (DMSO)-*d*_6_ with TMS as the internal standard, and chemical shifts are given in ppm (*δ*-scale). The MS spectra were recorded on a Thermo-Finnigan Trace DSQ GC MS apparatus (Waltham, Massachusetts, US). Chemicals were purchased from Merck Co., or Lancaster and used without further purification.

The purity of the obtained compounds was checked by TLC on aluminum oxide 60 F_254_ plates (Merck Co., Whitehouse Station, New Jersey, US), in a CHCl_3_/C_2_H_5_OH (10:1, v/v) solvent system with UV visualization (λ = 254 nm).

Elemental analysis of the obtained compounds was performed for C, H, N, S. The maximum percentage differences between calculated and found values for each element were within the error and amounted to ±0.4 %.

#### Crystal data for **2**

C_18_H_17_N_3_O_2_S, colorless prism, 0.45 × 0.29 × 0.14 mm^3^, monoclinic, *P*2_1_/*n*, *a* = 11.692(1) Å, *b* = 9.414(1) Å, *c* = 15.740(2) Å, *β* = 100.24(1)°, *V* = 1,704.9(3) Å^3^, *Z* = 4, *d*_calc_ = 1.322 g cm^−3^, *μ* = 0.205 mm^−1^, GooF = 0.977, data/restraints/parameters 3930/0/217 (*R*_int_ = 0.04), final *R* indices (*I* > 2*σ*(*I*)): *R*_1_ = 0.0548, *wR*_2_ = 0.0888, *R* indices (all data): *R*_1_ = 0.1867, *wR*_2_ = 0.1202, largest diff. peak and hole: 0.16 and −0.17 e Å^−3^.

Single-crystal diffraction data were measured at room temperature on an Oxford Diffraction Xcalibur diffractometer with the graphite-monochromated Mo K*α* radiation (λ = 0.71073). The programs CrysAlis CCD and CrysAlis Red (Oxford Diffraction, Xcalibur CCD System, 2006) were used for data collection, cell refinement, and data reduction. The intensity data were corrected for Lorentz and polarization effects. The structure was solved by direct methods using SHELXS-97 and refined by the full-matrix least-squares on *F* ^2^ using the SHELXL-97 (Sheldrick, 2008). All non-hydrogen atoms were refined with anisotropic displacement parameters. All H-atoms were positioned geometrically and allowed to ride on their parent atoms with *U*_iso_(H) = 1.2 *U*_eq_(C).

Crystallographic data have been deposited with the CCDC, 12 Union Road, Cambridge, CB2 1EZ, UK (fax: +44 1223 366033; e-mail: deposit@ccdc.cam.ac.uk or http://www.ccdc.cam.ac.uk) and are available on request, quoting the deposition number CCDC 860357.

### Ethyl 2-[(4,5-diphenyl-4*H*-1,2,4-triazol-3-yl)sulfanyl]acetate (**2**)

#### Method A

0.23 g (10 mmol) of sodium was added to 5 mL of anhydrous ethanol. The solution was placed in a three-necked flask equipped with reflux condenser and closed with a tube of CaCl_2_ and mercury stirred. The content was mixed till the sodium dissolved completely and then 2.53 g (10 mmol) of 4,5-diphenyl-4*H*-1,2,4-triazole-3-thione (**1**) was added. Then, 1.22 mL ethyl bromoacetate was added drop by drop. The content of the flask was mixed for 4 h and left at room temperature for 12 h. Then, 10 mL of anhydrous ethanol was added and heated for 1 h. The mixture was filtered of inorganic compounds. After cooling, the precipitate was filtered and crystallized from ethanol.

#### Method B

2.53 g (10 mmol) of 4,5-diphenyl-4*H*-1,2,4-triazole-3-thione (**1**) was dissolved in 10 mL of *N*,*N*-dimethylformamide. Then, 1 g of potassium carbonate and 1.22 mL of ethyl bromoacetate were added to the solution. The content of the flask was refluxed for 2 h. The mixture was filtered of inorganic compounds. Then, the distilled water was added and the precipitated compound was filtered, dried, and crystallized from ethanol.

Yield: 67.8 %, mp: 92–94 °C (dec.). Analysis for C_18_H_17_N_3_O_2_S (339.41); calculated: C, 63.70; H, 5.05; N, 12.38; S, 9.45; found: C, 63.92; H, 5.03; N, 12.41; S, 9.48. IR (KBr), *ν* (cm^−1^): 3091 (CH aromatic), 2955, 1422 (CH aliphatic), 1701 (C=O), 1611 (C=N), 676 (C–S). ^1^H NMR (DMSO-*d*_6_) *δ* (ppm): 1.19 (t, *J* = 6 Hz, 3H, CH_3_), 4.09 (s, 2H, CH_2_), 4.11–4.17 (q, *J* = 5 Hz, *J* = 5 Hz, 2H, CH_2_), 7.31–7.58 (m, 10H, 10ArH).

### [(4,5-Diphenyl-4*H*-1,2,4-triazol-3-yl)sulfanyl] acetohydrazide (**3**)

0.5 mL of 100 % hydrazine hydrate was added to 3.39 g (10 mmol) of compound **2** in 10 mL of anhydrous ethanol. The mixture was left at room temperature for 24 h. The precipitation of hydrazide **3** was filtered, dried, and crystallized from ethanol.

Yield: 91.4 %, mp: 196–198 °C (dec.). Analysis for C_16_H_15_N_5_OS (325.39); calculated: C, 59.06; H, 4.65; N, 21.52; S, 9.82; found: C, 59.10; H, 4.63; N, 21.49; S, 9.78. IR (KBr), *ν* (cm^−1^): 3105 (CH aromatic), 2980, 1423 (CH aliphatic), 1698 (C=O), 1611 (C=N), 1522 (C–N), 699 (C–S). ^1^H NMR (DMSO-*d*_6_) *δ* (ppm): 3.91 (s, 2H, CH_2_), 4.31 (s, 2H, NH_2_), 7.31–7.57 (m, 10H, 10ArH), 9.40 (brs, 1H, NH).

### Derivatives of thiosemicarbazide (**4a**–**l**)

#### General method (for compounds **4a**–**l**)

A mixture of 3.25 g (10 mmol) of hydrazide (**3**) and 10 mmol appropriate isothiocyanate was heated in an oil bath at 50–110 °C for 8–20 h. The product was washed with diethyl ether to remove unreacted isothiocyanate. Then it was filtered, dried, and crystallized from ethanol **4a**–**c**, **d**, **g**–**l**, butanol **4e**, or methanol **4f**.

#### Method B (for compounds **4a**, **c**, **d**)

10 mmol of appropriate isothiocyanate was added to 3.25 g (10 mmol) of hydrazide **3** in 10 mL of anhydrous diethyl ether. The mixture, placed in a conical bulb, was mixed for 5 min and left in room temperature for 24 h. The precipitation of thiosemicarbazide **4a**, **c**, **d** was filtered, dried, and crystallized from ethanol. The obtained compounds had the same melting points as the compounds obtained by the general method.

##### 4-Ethyl-1-{[(4,5-diphenyl-4*H*-1,2,4-triazol-3-yl)sulfanyl]acetyl} thiosemicarbazide (**4a**)

Yield: 94.0 %. Temperature of reaction: 70 °C for 8 h, mp: 205–207 °C (dec.). Analysis for C_19_H_20_N_6_OS_2_ (412.53); calculated: C, 55.32; H, 4.89; N, 20.37; S, 15.54; found: C, 55.23; H, 4.88; N, 20.43; S, 15.59. IR (KBr), *ν* (cm^−1^): 3199 (NH), 3101 (CH aromatic), 2974, 1453, 741 (CH aliphatic), 1699 (C=O), 1607 (C=N), 1519 (C–N), 1329 (C=S), 691 (C–S). ^1^H NMR (DMSO-*d*_6_) *δ* (ppm): 1.12 (t, *J* = 9 Hz, 3H, CH_3_), 3.51–3.60 (q, *J* = 7.5 Hz, *J* = 7.5 Hz, 2H, CH_2_), 3.90 (s, 2H, CH_2_), 7.34–7.57 (m, 10H, 10ArH), 8.32, 9.33, 10.25 (3brs, 3H, 3NH). ^13^C NMR *δ* (ppm): 14.61 (CH_3_), 30.75 (–S–CH_2_–), 33.90 (–CH_2_–CH_3_), 126.42, 127.68, 127.95, 128.79, 130.07, 130.11 (10CH aromatic), 130.33, 133.65 (2C aromatic), 152.08 (C–S), 154.59 (C-3 triazole), 166.82 (C=O), 181.23 (C=S). MS *m*/*z* (%): 412 (M^+^, 2), 397 (3), 335 (2), 325 (5), 294 (26), 253 (61), 252 (100), 194 (21), 180 (20), 149 (20), 118 (23), 104 (25), 91 (44), 77 (79).

##### 4-Allyl-1-{[(4,5-diphenyl-4*H*-1,2,4-triazol-3-yl)sulfanyl]acetyl} thiosemicarbazide (**4b**)

Yield: 90.7 %. Temperature of reaction: 55 °C for 12 h, mp: 192–194 °C (dec.). Analysis for C_20_H_20_N_6_OS_2_ (424.54); calculated: C, 56.58; H, 4.75; N, 19.79; S, 15.10; found: C, 56.53; H, 4.76; N, 19.81; S, 15.14. IR (KBr), *ν* (cm^−1^): 3218 (NH), 3078 (CH aromatic), 2963, 1431, 761 (CH aliphatic), 1705 (C=O), 1603 (C=N), 1511 (C–N), 1351 (C=S), 686 (C–S). ^1^H NMR (DMSO-*d*_6_) *δ* (ppm): 3.95 (s, 2H, CH_2_), 4.13 (d, *J* = 5 Hz, 2H, CH_2_), 5.02–5.13 (dd, *J* = 5 Hz, *J* = 5 Hz, 2H, =CH_2_), 5.79–5.90 (m, 1H, CH), 7.35–7.57 (m, 10H, 10ArH), 8.45, 9.45, 10.33 (3brs, 3H, 3NH).

##### 4-Cyclohexyl-1-{[(4,5-diphenyl-4*H*-1,2,4-triazol-3-yl)sulfanyl]acetyl} thiosemicarbazide (**4c**)

Yield: 64.5 %. Temperature of reaction: 50 °C for 12 h, mp: 188–190 °C (dec.). Analysis for C_23_H_26_N_6_OS_2_ (466.62); calculated: C, 59.20; H, 5.62; N, 18.01; S, 13.74; found: C, 59.35; H, 5.63; N, 17.95; S, 13.70. IR (KBr), *ν* (cm^−1^): 3208 (NH), 3109 (CH aromatic), 2987, 1424, 753 (CH aliphatic), 1699 (C=O), 1595 (C=N), 1519 (C–N), 1331 (C=S), 689 (C–S). ^1^H NMR (DMSO-*d*_6_) *δ* (ppm): 1.01–1.72 (m, 10H, 5CH_2_ cyclohexane), 3.87 (s, 2H, CH_2_), 4.31 (m, 1H, CH cyclohexane), 7.28–7.56 (m, 10H, 10ArH), 8.71, 9.35, 10.20 (3brs, 3H, 3NH).

##### 4-Phenyl-1-{[(4,5-diphenyl-4*H*-1,2,4-triazol-3-yl)sulfanyl]acetyl} thiosemicarbazide (**4d**)

Yield: 91.0 %. Temperature of reaction: 50 °C for 15 h, mp: 178–180 °C (dec.). Analysis for C_23_H_20_N_6_OS_2_ (460.57); calculated: C, 59.98; H, 4.38; N, 18.25; S, 13.92; found: C, 60.03; H, 4.38; N, 18.30; S, 13.96. IR (KBr), *ν* (cm^−1^): 3205 (NH), 3114 (CH aromatic), 2978 (CH aliphatic), 1705 (C=O), 1610 (C=N), 1516 (C–N), 1337 (C=S), 685 (C–S). ^1^H NMR (DMSO-*d*_6_) *δ* (ppm): 4.00 (s, 2H, CH_2_), 7.12–7.51 (m, 15H, 15ArH), 9.38, 9.76, 10.47 (3brs, 3H, 3NH). ^13^C NMR *δ* (ppm): 34.55 (–S–CH_2_–), 125.23, 125.79, 126.45, 127.77, 127.92, 128.09, 128.75, 130.07, 130.15 (15CH aromatic), 130.36, 133.78, 139.09 (3C aromatic), 151.75 (C–S), 154.48 (C-3 triazole), 166.95 (C=O), 180.98 (C=S). MS *m*/*z* (%): 460 (M^+^, 1), 383 (1.2), 325 (13), 294 (20), 252 (60), 194 (10), 180 (10), 149 (8), 135 (74), 131 (5), 104 (25), 91 (33), 77 (100).

##### 4-(4-Bromophenyl)-1-{[(4,5-diphenyl-4*H*-1,2,4-triazol-3-yl)sulfanyl]acetyl} thiosemicarbazide (**4e**)

Yield: 88.3 %. Temperature of reaction: 110 °C for 16 h, mp: 188–190 °C (dec.). Analysis for C_23_H_19_BrN_6_OS_2_ (539.47); calculated: C, 51.21; H, 3.55; N, 15.58; S, 11.88; Br, 14.81; found: C, 51.27; H, 3.54; N, 15.61; S, 11.92. IR (KBr), *ν* (cm^−1^): 3213 (NH), 3116 (CH aromatic), 2972 (CH aliphatic), 1703 (C=O), 1600 (C=N), 1341 (C=S), 690 (C–S). ^1^H NMR (DMSO-*d*_6_) *δ* (ppm): 3.97 (s, 2H, CH_2_), 7.29–7.55 (m, 14H, 14ArH), 9.79, 9.82, 10.46 (3brs, 3H, 3NH).

##### 4-(4-Chlorophenyl)-1-{[(4,5-diphenyl-4*H*-1,2,4-triazol-3-yl)sulfanyl]acetyl} thiosemicarbazide (**4f**)

Yield: 97.8 %.Temperature of reaction: 100 °C for 16 h, mp: 180–184 °C (dec.). Analysis for C_23_H_19_ClN_6_OS_2_ (495.02); calculated: C, 55.80; H, 3.87; N, 16.98; S, 12.95; Cl, 9.16; found: C, 55.83; H, 3.88; N, 16.93; S, 12.90. IR (KBr), *ν* (cm^−1^): 3202 (NH), 3093 (CH aromatic), 2983 (CH aliphatic), 1705 (C=O), 1608 (C=N), 1338 (C=S), 688 (C–S). ^1^H NMR (DMSO-*d*_6_) *δ* (ppm): 3.98 (s, 2H, CH_2_), 7.31–7.56 (m, 14H, 14ArH), 9.81, 9.88, 10.46 (3brs, 3H, 3NH). ^13^C NMR *δ* (ppm): 34.39 (–S–CH_2_–), 121.95, 125.63, 128.40, 128.56, 128.76, 129.37, 129.54, 130.08 (14CH aromatic), 128.60, 130.19, 133.65, 137.92 (4C aromatic), 151.63 (C–S), 154.30 (C-3 triazole), 166.85 (C=O), 180.84 (C=S).

##### 4-(4-Methoxyphenyl)-1-{[(4,5-diphenyl-4*H*-1,2,4-triazol-3-yl)sulfanyl]acetyl} thiosemicarbazide (**4g**)

Yield: 95.2 %.Temperature of reaction: 60 °C for 18 h, mp: 172–174 °C (dec.). Analysis for C_24_H_22_N_6_O_2_S_2_ (490.60); calculated: C, 58.75; H, 4.52; N, 17.13; S, 13.07; found: C, 58.97; H, 4.51; N, 17.18; S, 13.10. IR (KBr), *ν* (cm^−1^): 3198 (NH), 3102 (CH aromatic), 2988, 1452, 759 (CH aliphatic), 1710 (C=O), 1605 (C=N), 1519 (C–N), 1329 (C=S), 693 (C–S). ^1^H NMR (DMSO-*d*_6_) *δ* (ppm): 3.74 (s, 3H, CH_3_), 3.99 (s, 2H, CH_2_), 6.90 (d, *J* = 6 Hz, 2H, 2ArH), 7.32–7.56 (m, 10H, 10ArH), 7.57 (d, *J* = 6 Hz, 2H, 2ArH), 9.61, 9.66, 10.40 (3brs, 3H, 3NH).

##### 4-Benzyl-1-{[(4,5-diphenyl-4*H*-1,2,4-triazol-3-yl)sulfanyl]acetyl} thiosemicarbazide (**4h**)

Yield: 95.0 %. Temperature of reaction: 50 °C for 12 h, mp: 176–180 °C (dec.). Analysis for C_24_H_22_N_6_OS_2_ (474.60); calculated: C, 60.74; H, 4.67; N, 17.71; S, 13.51; found: C, 60.77; H, 4.66; N, 17.78; S, 13.55. IR (KBr), *ν* (cm^−1^): 3209 (NH), 3087 (CH aromatic), 2971, 1439 (CH aliphatic), 1700 (C=O), 1611 (C=N), 1520 (C–N), 1351 (C=S), 689 (C–S). ^1^H NMR (DMSO-*d*_6_) *δ* (ppm): 3.90 (s, 2H, CH_2_), 4.84 (s, 2H, CH_2_), 7.15–7.54 (m, 15H, 15ArH), 8.82, 9.54, 10.41 (3brs, 3H, 3NH). ^13^C NMR *δ* (ppm): 33.68 (–S–CH_2_–), 46.62 (–CH_2_–), 126.47, 127.12, 127.46, 127.83, 128.16, 128.51, 128.83, 129.83, 130.04 (15CH aromatic), 133.71, 134.71, 139.34 (3C aromatic), 151.95 (C–S), 154.32 (C-3 triazole), 166.79 (C=O), 182.09 (C=S).

##### 4-(4-Methoxybenzyl)-1-{[(4,5-diphenyl-4*H*-1,2,4-triazol-3-yl)sulfanyl]acetyl} thiosemicarbazide (**4i**)

Yield: 97.4 %. Temperature of reaction: 50 °C for 14 h, mp: 176–178 °C (dec.). Analysis for C_25_H_24_N_6_O_2_S_2_ (504.63); calculated: C, 59.50; H, 4.79; N, 16.65; S, 12.71; found: C, 59.61; H, 4.78; N, 16.68; S, 12.75. IR (KBr), *ν* (cm^−1^): 3222 (NH), 3102 CH (aromatic), 2973, 1448, 767 (CH aliphatic), 1697 (C=O), 1599 (C=N), 1514 (C–N), 1349 (C=S), 680 (C–S). ^1^H NMR (DMSO-*d*_6_) *δ* (ppm): 3.76 (s, 3H, CH_3_), 4.01 (s, 2H, CH_2_), 4.74 (s, 2H, CH_2_), 6.86–7.64 (m, 14H, 14ArH), 8.33, 9.55, 10.44 (3brs, 3H, 3NH).

##### 4-Ethoxycarbonyl-1-{[(4,5-diphenyl-4*H*-1,2,4-triazol-3-yl)sulfanyl]acetyl} thiosemicarbazide (**4j**)

Yield: 98.6 %. Temperature of reaction: 55 °C for 14 h, mp: 178–180 °C (dec.). Analysis for C_20_H_20_N_6_O_3_S_2_ (456.54); calculated: C, 52.62; H, 4.41; N, 18.41; S, 14.05; found: C, 52.76; H, 4.42; N, 18.44; S, 14.01. IR (KBr), *ν* (cm^−1^): 3219 (NH), 3105 (CH aromatic), 2973, 1452, 765 (CH aliphatic), 1728 (C=O acidic), 1699 (C=O), 1608 (C=N), 1511 (C–N), 1338 (C=S), 691 (C–S). ^1^H NMR (DMSO-*d*_6_) *δ* (ppm): 1.22 (t, *J* = 5 Hz, 3H, CH_3_), 4.09 (s, 2H, CH_2_), 4.12–4.21 (q, *J* = 7.5 Hz, *J* = 7.5 Hz, 2H, CH_2_), 7.28–7.56 (m, 10H, 10ArH), 11.07, 11.38, 11.51 (3brs, 3H, 3NH).

##### 4-Ethoxycarbonylmethyl-1-{[(4,5-diphenyl-4*H*-1,2,4-triazol-3-yl)sulfanyl]acetyl} thiosemicarbazide (**4k**)

Yield: 91.9 %. Temperature of reaction: 50 °C for 14 h, mp: 188–190 °C (dec.). Analysis for C_21_H_22_N_6_O_3_S_2_ (470.57); calculated: C, 53.60; H, 4.71; N, 17.86; S, 13.63; found: C, 53.46; H, 4.72; N, 17.90; S, 13.67. IR (KBr), *ν* (cm^−1^): 3211 (NH), 3096 (CH aromatic), 2975, 1464, 758 (CH aliphatic), 1737 (C=O acidic), 1703 (C=O), 1611 (C=N), 1511 (C–N), 1344 (C=S), 686 (C–S). ^1^H NMR (DMSO-*d*_6_) *δ* (ppm): 1.16 (t, *J* = 5 Hz, 3H, CH_3_), 3.96 (s, 2H, CH_2_), 4.02–4.11 (q, *J* = 7.5 Hz, *J* = 7.5 Hz, 2H, CH_2_), 4.30 (s, 2H, CH_2_), 7.33–7.58 (m, 10H, 10ArH), 8.78, 9.69, 10.47 (3brs, 3H, 3NH).

##### 4-Benzoyl-1-{[(4,5-diphenyl-4*H*-1,2,4-triazol-3-yl)sulfanyl]acetyl} thiosemicarbazide (**4l**)

Yield: 96.8 %. Temperature of reaction: 50 °C for 20 h, mp: 180–182 °C (dec.). Analysis for C_24_H_20_N_6_O_2_S_2_ (488.58); calculated: C, 59.00; H, 4.13; N, 17.20; S, 13.12; found: C, 58.95; H, 4.12; N, 17.26; S, 13.08. IR (KBr), *ν* (cm^−1^): 3176 (NH), 3088 (CH aromatic), 2979, 1449 (CH aliphatic), 1746 (C=O acidic), 1703 (C=O), 1608 (C=N), 1509 (C–N), 1311 (C=S), 681 (C–S). ^1^H NMR (DMSO-*d*_6_) *δ* (ppm): 4.15 (s, 2H, CH_2_), 7.35–7.96 (m, 15H, 15ArH), 11.33, 11.77, 12.87 (3brs, 3H, 3NH).

### Derivatives of 4,5-disubstituted-1,2,4-triazole-3(2*H*)-thione (**5a**–**i**)

#### General procedure

A mixture of thiosemicarbazide **4a**–**i** (10 mmol) and 20–40 mL of 2 % aqueous solution of sodium hydroxide was refluxed for 2 h. Then, the solution was neutralized with diluted hydrochloric acid and the formed precipitate was filtered and crystallized from ethanol **5c**, **d**, **h**, **i**, butanol **5b**, **e**, **f**, or methanol **5a**, **g**.

##### 4-Ethyl-5-{[(4,5-diphenyl-4*H*-1,2,4-triazol-3-yl)sulfanyl]methyl}-4*H*-1,2,4-triazole-3(2*H*)-thione (**5a**)

Yield: 87.6 %, mp: 214–216 °C (dec.). Analysis for C_19_H_18_N_6_S_2_ (394.52); calculated: C, 57.84; H, 4.60; N, 21.30; S, 16.25; found: C, 57.67; H, 4.59; N, 21.33; S, 16.21. IR (KBr), *ν* (cm^−1^): 3135 (NH), 3085 (CH aromatic), 2958, 1422, 758 (CH aliphatic), 1600 (C=N), 1502 (C–N), 1350 (C=S), 692 (C–S). ^1^H NMR (DMSO-*d*_6_) *δ* (ppm): 1.22 (t, *J* = 5 Hz, 3H, CH_3_), 3.91–3.97 (q, *J* = 5 Hz, *J* = 5 Hz, 2H, CH_2_), 4.39 (s, 2H, CH_2_), 7.27–7.54 (m, 10H, 10ArH), 13.62 (s, 1H, NH). MS *m*/*z* (%): 394 (M^+^, 0.2), 365 (0.1), 339 (0.12), 264 (0.1), 253 (64), 252 (68), 194 (21), 149 (33), 128 (16), 118 (37), 104 (10), 91 (58), 77 (100).

##### 4-Allyl-5-{[(4,5-diphenyl-4*H*-1,2,4-triazol-3-yl)sulfanyl]methyl}-4*H*-1,2,4-triazole-3(2*H*)-thione (**5b**)

Yield: 90.5 %, mp: 207–208 °C (dec.). Analysis for C_20_H_18_N_6_S_2_ (406.53); calculated: C, 59.10; H, 4.46; N, 20.67; S, 15.77; found: C, 58.96; H, 4.45; N, 20.64; S, 15.74. IR (KBr), *ν* (cm^−1^): 3185 (NH), 3091 (CH aromatic), 2989, 1450, 756 (CH aliphatic), 1604 (C=N), 1510 (C–N), 1343 (C=S), 684 (C–S). ^1^H NMR (DMSO-*d*_6_) *δ* (ppm): 4.44 (s, 2H, CH_2_), 4.69–4.71 (d, *J* = 5 Hz, 2H, CH_2_), 5.24–5.41 (dd, *J* = 5 Hz, *J* = 5 Hz, 2H, =CH_2_), 5.82–5.93 (m, 1H, CH), 7.37–7.62 (m, 10H, 10ArH), 13.81 (brs, 1H, NH).

##### 4-Cyclohexyl-5-{[(4,5-diphenyl-4*H*-1,2,4-triazol-3-yl)sulfanyl]methyl}-4*H*-1,2,4-triazole-3(2*H*)-thione (**5c**)

Yield: 62.4 %, mp: 186–188 °C (dec.). Analysis for C_23_H_24_N_6_S_2_ (448.61); calculated: C, 61.58; H, 5.39; N, 18.73; S, 14.29; found: C, 61.37; H, 5.38; N, 18.68; S, 14.32. IR (KBr), *ν* (cm^−1^): 3175 (NH), 3088 (CH aromatic), 2963, 1449, 759 (CH aliphatic), 1611 (C=N), 1505 (C–N), 1339 (C=S), 684 (C–S). ^1^H NMR (DMSO-*d*_6_) *δ* (ppm): 1.05–1.73 (m, 10H, 5CH_2_ cyclohexane), 4.04 (s, 2H, CH_2_), 4.45 (m, 1H, CH cyclohexane), 7.29–7.56 (m, 10H, 10ArH), 14.13 (brs, 1H, NH).

##### 4-Phenyl-5-{[(4,5-diphenyl-4*H*-1,2,4-triazol-3-yl)sulfanyl]methyl}-4*H*-1,2,4-triazole-3(2*H*)-thione (**5d**)

Yield: 76.9 %, mp: 209–210 °C (dec.). Analysis for C_23_H_18_N_6_S_2_ (442.56); calculated: C, 62.42; H, 4.10; N, 18.99; S, 14.49; found: C, 62.28; H, 4.09; N, 18.93; S, 14.51. IR (KBr), *ν* (cm^−1^): 3175 (NH), 3090 (CH aromatic), 2972 (CH aliphatic), 1598 (C=N), 1505 (C–N), 1326 (C=S), 684 (C–S). ^1^H NMR (DMSO-*d*_6_) *δ* (ppm): 4.14 (s, 2H, CH_2_), 7.12–7.59 (m, 15H, 15ArH), 13.86 (brs, 1H, NH). ^13^C NMR *δ* (ppm): 26.22 (–S–CH_2_–), 125.61, 128.44, 128.55, 128.63, 128.74, 129.23, 129.41, 129.58, 130.11 (15CH aromatic), 138.23, 146.83, 148.15 (3C aromatic), 150.65 (C-3′ triazole), 153.33 (C–S), 166.98 (C-3 triazole), 167.42 (C=S). MS *m*/*z* (%): 442 (M^+^, 2), 306 (1), 294 (1), 252 (98), 194 (23), 149 (18), 127 (14), 118 (44), 104 (8), 91 (27), 77 (100).

##### 4-(4-Bromophenyl)-5-{[(4,5-diphenyl-4*H*-1,2,4-triazol-3-yl)sulfanyl]methyl}-4*H*-1,2,4-triazole-3(2*H*)-thione (**5e**)

Yield: 97.2 %, mp: 210–212 °C (dec.). Analysis for C_23_H_17_BrN_6_S_2_ (521.45); calculated: C, 52.98; H, 3.29; N, 16.12; S, 12.30; Br, 15.32; found: C, 52.93; H, 3.28; N, 16.15; S, 12.32. IR (KBr), *ν* (cm^−1^): 3178 (NH), 3102 (CH aromatic), 2965, 1448 (CH aliphatic), 1609 (C=N), 1504 (C–N), 1367 (C=S), 688 (C–S). ^1^H NMR (DMSO-*d*_6_) *δ* (ppm): 4.17 (s, 2H, CH_2_), 7.14–7.46 (m, 14H, 14ArH), 13.89 (brs, 1H, NH).

##### 4-(4-Chlorophenyl)-5-{[(4,5-diphenyl-4*H*-1,2,4-triazol-3-yl)sulfanyl]methyl}-4*H*-1,2,4-triazole-3(2*H*)-thione (**5f**)

Yield: 96.0 %, mp: 118–120 °C (dec.). Analysis for C_23_H_17_ClN_6_S_2_ (477.00); calculated: C, 57.91; H, 3.59; N, 17.62; S, 13.44; Cl, 7.43; found: C, 57.85; H, 3.58; N, 17.65; S, 13.41. IR (KBr), *ν* (cm^−1^): 3143 (NH), 3088 (CH aromatic), 2985, 1459 (CH aliphatic), 1601 (C=N), 1500 (C–N), 1361 (C=S), 690 (C–S). ^1^H NMR (DMSO-*d*_6_) *δ* (ppm): 4.17 (s, 2H, CH_2_), 7.22–7.58 (m, 14H, 14ArH), 13.89 (brs, 1H, NH).

##### 4-(4-Methoxyphenyl)-5-{[(4,5-diphenyl-4*H*-1,2,4-triazol-3-yl)sulfanyl]methyl}-4*H*-1,2,4-triazole-3(2*H*)-thione (**5g**)

Yield: 98.3 %, mp: 206–208 °C (dec.). Analysis for C_24_H_20_N_6_OS_2_ (472.58); calculated: C, 60.99; H, 4.26; N, 17.78; S, 13.57; found: C, 61.16; H, 4.25; N, 17.71; S, 13.61. IR (KBr), *ν* (cm^−1^): 3164 (NH), 3094 (CH aromatic), 2969, 1441 (CH aliphatic), 1612 (C=N), 1506 (C–N), 1319 (C=S), 691 (C–S). ^1^H NMR (DMSO-*d*_6_) *δ* (ppm): 3.80 (s, 3H, CH_3_), 4.14 (s, 2H, CH_2_), 7.03–7.59 (m, 14H, 14ArH), 13.82 (brs, 1H, NH). ^13^C NMR *δ* (ppm): 27.55 (–S–CH_2_–), 55.56 (CH_3_), 114.56, 125.67, 127.62, 128.33, 128.67, 129.32, 129.55, 130.11 (14CH aromatic), 133.71, 134.62, 139.61, 159.81 (4C aromatic), 150.51 (C-3′ triazole), 154.73 (C–S), 168.42 (C-3 triazole), 168.61 (C=S). MS *m*/*z* (%): 472 (M^+^, 0.07), 457 (0.03), 440 (0.02), 339 (0.21), 306 (0.05), 294 (0.11), 268 (11), 252 (59), 209 (7), 194 (15), 149 (12), 135 (86), 118 (13), 104 (10), 91 (27), 77 (100).

##### 4-Benzyl-5-{[(4,5-diphenyl-4*H*-1,2,4-triazol-3-yl)sulfanyl]methyl}-4*H*-1,2,4-triazole-3(2*H*)-thione (**5h**)

Yield: 79.0 %, mp: 136–140 °C (dec.). Analysis for C_24_H_20_N_6_S_2_ (456.58); calculated: C, 63.13; H, 4.41; N, 18.41; S, 14.04; found: C, 63.26; H, 4.42; N, 18.35; S, 14.08. IR (KBr), *ν* (cm^−1^): 3155 (NH), 3091 (CH aromatic) 2961, 1453, 762 (CH aliphatic), 1609 (C=N), 1508 (C–N), 1342 (C=S), 677 (C–S). ^1^H NMR (DMSO-*d*_6_) *δ* (ppm): 4.29 (s, 2H, CH_2_), 5.24 (s, 2H, CH_2_), 7.22–7.53 (m, 15H, 15ArH), 13.86 (brs, 1H, NH).

##### 4-(4-Methoxybenzyl)-5-{[(4,5-diphenyl-4*H*-1,2,4-triazol-3-yl)sulfanyl]methyl}-4*H*-1,2,4-triazole-3(2*H*)-thione (**5i**)

Yield: 98.5 %, mp: 118–120 °C (dec.). Analysis for C_25_H_22_N_6_OS_2_ (486.61); calculated: C, 61.70; H, 4.56; N, 17.27; S, 13.18; found: C, 61.61; H, 4.55; N, 17.25; S, 13.14. IR (KBr), *ν* (cm^−1^): 3174 (NH), 3071 (CH aromatic), 2982, 1453, 764 (CH aliphatic), 1612 (C=N), 1510 (C–N), 1358 (C=S), 673 (C–S). ^1^H NMR (DMSO-*d*_6_) *δ* (ppm): 3.71 (s, 3H, CH_3_), 4.33 (s, 2H, CH_2_), 5.20 (s, 2H, CH_2_), 6.83–7.52 (m, 14H, 14ArH), 13.82 (brs, 1H, NH).

### Derivatives of 2,5-disubstituted-1,3,4-thiadiazole (**6a**–**i**)

#### Method A (for compounds **6a**–**i**)

10 mmol of 4-substituted-1-{[(4,5-diphenyl-4*H*-1,2,4-triazol-3-yl)sulfanyl]acetyl} thiosemicarbazide **4a**–**i** was dissolved in 10–20 mL diluted sulfuric acid and stirred in a closed bulb for 1 h. Subsequently, the solution was poured out on crushed ice (50 g) and stirred until the ice was completely dissolved. Later, the solution was neutralized with ammonium hydroxide. The precipitate that formed was filtered, dried, and crystallized from ethanol **6a**, **c**, **d**, **g**–**i** or butanol **6b**, **e**, **f**.

#### Method B (for compounds **6a**, **d**)

20 mL of 10 % ethanolic solution of hydrochloric acid was added to thiosemicarbazide **4a**, **d** and the reaction mixture was heated under reflux for 1 h. Subsequently, the solution was left at room temperature for 24 h. The precipitate formed was separated by filtration, dried, and crystallized from ethanol.

#### Method C (for compounds **6e**, **f**)

A mixture of 10 mmol of thiosemicarbazide **4e**, **f** in 10 mL of anhydrous acetic acid was refluxed for 1 h. Subsequently, the solution was left at room temperature for 12 h. The precipitate that formed was separated by filtration, dried, and crystallized from butanol.

##### 5-Aminoethyl-2-{[(4,5-diphenyl-4*H*-1,2,4-triazol-3-yl)sulfanyl]methyl}-1,3,4-thiadiazole (**6a**)

Yield: 81.3 %, mp: 168–170 °C (dec.). Analysis for C_19_H_18_N_6_S_2_ (394.52); calculated: C, 57.84; H, 4.60; N, 21.30; S, 16.25; found: C, 57.69; H, 4.58; N, 21.26; S, 16.21. IR (KBr), *ν* (cm^−1^): 3244 (NH), 3071 (CH aromatic), 2944, 1458, 733 (CH aliphatic), 1602 (C=N), 1506 (C–N), 671 (C–S). ^1^H NMR (DMSO-*d*_6_) *δ* (ppm): 1.13 (t, *J* = 7.5 Hz, 3H, CH_3_), 3.21–3.27 (q, *J* = 5 Hz, *J* = 5 Hz, 2H, CH_2_), 4.57 (s, 2H, CH_2_), 7.17–7.70 (m, 10H, 10ArH), 9.35 (brs, 1H, NH).

##### 5-Aminoallyl-2-{[(4,5-diphenyl-4*H*-1,2,4-triazol-3-yl)sulfanyl]methyl}-1,3,4-thiadiazole (**6b**)

Yield: 68.9 %, mp: 208–210 °C (dec.). Analysis for C_20_H_18_N_6_S_2_ (406.53); calculated: C, 59.09; H, 4.46; N, 20.67; S, 15.77; found: C, 59.22; H, 4.45; N, 20.65; S, 15.73. IR (KBr), *ν* (cm^−1^): 3256 (NH), 3083 (CH aromatic), 2955, 1489, 741 (CH aliphatic), 1610 (C=N), 1503 (C–N), 679 (C–S). ^1^H NMR (DMSO-*d*_6_) *δ* (ppm): 3.87 (s, 2H, CH_2_), 4.12 (d, *J* = 5 Hz, 2H, CH_2_), 5.02–5.13 (dd, *J* = 5 Hz, *J* = 5 Hz, 2H, =CH_2_), 5.79–5.88 (m, 1H, CH), 7.40–8.56 (m, 10H, 10ArH), 10.13 (brs, 1H, NH).

##### 5-Aminocyclohexyl-2-{[(4,5-diphenyl-4*H*-1,2,4-triazol-3-yl)sulfanyl]methyl}-1,3,4-thiadiazole (**6c**)

Yield: 75.6 %, mp: 172–174 °C (dec.). Analysis for C_23_H_24_N_6_S_2_ (448.61); calculated: C, 61.58; H, 5.39; N, 18.73; S, 14.30; found: C, 61.61; H, 5.37; N, 18.76; S, 14.27. IR (KBr), *ν* (cm^−1^): 3190 (NH), 3093 (CH aromatic), 2972, 1467, 749 (CH aliphatic), 1620 (C=N), 681 (C–S). ^1^H NMR (DMSO-*d*_6_) *δ* (ppm): 1.1–1.65 (m, 10H, 5CH_2_ cyclohexane), 3.03 (m, 1H, CH cyclohexane), 4.22 (s, 2H, CH_2_), 7.33–8.06 (m, 10H, 10ArH), 10.16 (brs, 1H, NH).

##### 5-Aminophenyl-2-{[(4,5-diphenyl-4*H*-1,2,4-triazol-3-yl)sulfanyl]methyl}-1,3,4-thiadiazole (**6d**)

Yield: 50.9 %, mp: 192–198 °C (dec.). Analysis for C_23_H_18_N_6_S_2_ (442.60); calculated: C, 62.42; H, 4.10; N, 19.00; S, 14.49; found: C, 62.36; H, 4.09; N, 18.97; S, 14.53. IR (KBr), *ν* (cm^−1^): 3199 (NH), 3011 (CH aromatic), 2968 (CH aliphatic), 1610 (C=N), 1504 (C–N), 683 (C–S). ^1^H NMR (DMSO-*d*_6_) *δ* (ppm): 4.02 (s, 2H, CH_2_), 6.98–7.54 (m, 15H, 15ArH), 10.42 (brs, 1H, NH).

##### [5-Amino-(4-bromophenyl)]-2-{[(4,5-diphenyl-4*H*-1,2,4-triazol-3-yl)sulfanyl]methyl}-1,3,4-thiadiazole (**6e**)

Yield: 89.4 %, mp: 203–205 °C (dec.). Analysis for C_23_H_17_BrN_6_S_2_ (521.45); calculated: C, 52.98; H, 3.29; N, 16.12; S, 12.30; Br, 15.32; found: C, 52.73; H, 3.27; N, 16.15; S, 12.27. IR (KBr), *ν* (cm^−1^): 3167 (NH), 3110 (CH aromatic), 2954, 1441 (CH aliphatic), 1602 (C=N), 680 (C–S). ^1^H NMR (DMSO-*d*_6_) *δ* (ppm): 4.22 (s, 2H, CH_2_), 6.89–7.65 (m, 14H, 14ArH), 10.23 (brs, 1H, NH).

##### [5-Amino-(4-chlorophenyl)]-2-{[(4,5-diphenyl-4*H*-1,2,4-triazol-3-yl)sulfanyl]methyl}-1,3,4-thiadiazole (**6f**)

Yield: 94.7 %, mp: 215–218 °C (dec.). Analysis for C_23_H_17_ClN_6_S_2_ (477.00); calculated: C, 57.91; H, 3.59; N, 17.62; S, 13.44; Cl, 7.43; found: C, 57.71; H, 3.60; N, 17.58; S, 13.39. IR (KBr), *ν* (cm^−1^): 3245 (NH), 3065 (CH aromatic), 2977 (CH aliphatic), 1611 (C=N), 1506 (C–N), 695 (C–S). ^1^H NMR (DMSO-*d*_6_) *δ* (ppm): 3.89 (s, 2H, CH_2_), 7.39–7.64 (m, 14H, 14ArH), 10.36 (brs, 1H, NH).

##### [5-Amino-(4-methoxyphenyl)]-2-{[(4,5-diphenyl-4*H*-1,2,4-triazol-3-yl)sulfanyl]methyl}-1,3,4-thiadiazole (**6g**)

Yield: 53.6 %, mp: 152–154 °C (dec.). Analysis for C_24_H_20_N_6_OS_2_ (472.58); calculated: C, 60.99; H, 4.26; N, 17.78; S, 13.57; found: C, 60.89; H, 4.26; N, 17.75; S, 14.55. IR (KBr), *ν* (cm^−1^): 3211 (NH), 3038 (CH aromatic), 2982, 1451 (CH aliphatic), 1600 (C=N), 1502 (C–N), 692 (C–S). ^1^H NMR (DMSO-*d*_6_) *δ* (ppm): 3.71 (s, 3H, CH_3_), 4.65 (s, 2H, CH_2_), 6.89–7.78 (m, 14H, 14ArH), 10.07 (brs, 1H, NH).

##### 5-Aminobenzyl-2-{[(4,5-diphenyl-4*H*-1,2,4-triazol-3-yl)sulfanyl]methyl}-1,3,4-thiadiazole (**6h**)

Yield: 70.2 %, mp: 146–148 °C (dec.). Analysis for C_24_H_20_N_6_S_2_ (456.58); calculated: C, 63.13; H, 4.41; N, 18.41; S, 14.04; found: C, 63.05; H, 4.39; N, 18.36; S, 14.09. IR (KBr), *ν* (cm^−1^): 3272 (NH), 3042 (CH aromatic), 2934, 1458 (CH aliphatic), 1601 (C=N), 1512 (C–N), 686 (C–S). ^1^H NMR (DMSO-*d*_6_) *δ* (ppm): 4.11 (s, 2H, CH_2_), 4.73 (s, 2H, CH_2_), 7.34–7.62 (m, 15H, 15ArH), 10.47 (brs, 1H, NH).

##### [5-Amino-(4-methoxybenzyl)]-2-{[(4,5-diphenyl-4*H*-1,2,4-triazol-3-yl)sulfanyl]methyl}-1,3,4-thiadiazole (**6i**)

Yield: 71.4 %, mp: 218–220 °C (dec.). Analysis for C_25_H_22_N_6_OS_2_ (486.61); calculated: C, 61.70; H, 4.56; N, 17.27; S, 13.18; found: C, 61.77; H, 4.55; N, 17.23; S, 13.22. IR (KBr), *ν* (cm^−1^): 3268 (NH), 3095 (CH aromatic), 2955, 1420, 765 (CH aliphatic), 1598 (C=N), 1508 (C–N), 690 (C–S). ^1^H NMR (DMSO-*d*_6_) *δ* (ppm): 3.68 (s, 3H, CH_3_), 3.98 (s, 2H, CH_2_), 4.44 (s, 2H, CH_2_), 6.86–7.64 (m, 14H, 14ArH), 10.44 (brs, 1H, NH).

### Derivatives of *N*,*N*-disubstituted acetamide (**7a**–**i**)

#### General method (for compounds **7a**–**i**)

A mixture of 10 mmol of appropriate 2,5-disubstituted-1,3,4-thiadiazole **6a**–**i** in 5 mL of acetic anhydride was heated under reflux for 2 h. Distilled water was added to the reaction mixture and it was allowed to cool. The resulting precipitate was filtered and washed with distilled water. The residue was purified by recrystallization from ethanol.

##### *N*-(5-{[(4,5-diphenyl-4*H*-1,2,4-triazol-3-yl)sulfanyl]methyl}-1,3,4-thiadiazol-2-yl)-*N*-ethylacetamide (**7a**)

Yield: 75.6 %, mp: 182–184 °C (dec.). Analysis for C_21_H_20_N_6_OS_2_ (436.55); calculated: C, 57.78; H, 4.62; N, 19.25; S, 14.69; found: C, 57.81; H, 4.61; N, 19.28; S, 14.69. IR (KBr), *ν* (cm^−1^): 3091 (CH aromatic), 2922, 1467, 742 (CH aliphatic), 1701 (C=O), 1610 (C=N), 1512 (C–N), 692 (C–S). ^1^H NMR (DMSO-*d*_6_) *δ* (ppm): 1.31 (t, *J* = 7.5 Hz, 3H, CH_3_), 2.15 (s, 3H, CH_3_), 3.65–3.70 (q, *J* = 5 Hz, *J* = 5 Hz, 2H, CH_2_), 4.44 (s, 2H, CH_2_), 7.33–8.04 (m, 10H, 10ArH).

##### *N*-(5-{[(4,5-diphenyl-4*H*-1,2,4-triazol-3-yl)sulfanyl]methyl}-1,3,4-thiadiazol-2-yl)-*N*-allylacetamide (**7b**)

Yield: 62.1 %, mp: 212–214 °C (dec.). Analysis for C_22_H_20_N_6_OS_2_ (448.56); calculated: C, 58.91; H, 4.49; N, 18.74; S, 14.30; found: C, 58.94; H, 4.51; N, 18.76; S, 14.28. IR (KBr), *ν* (cm^−1^): 3122 (CH aromatic), 2978, 1492, 742 (CH aliphatic), 1708 (C=O), 1614 (C=N), 1515 (C–N), 688 (C–S). ^1^H NMR (DMSO-*d*_6_) *δ* (ppm): 2.11 (s, 3H, CH_3_), 4.27 (s, 2H, CH_2_), 4.35 (d, *J* = 5 Hz, 2H, CH_2_), 5.14–5.18 (dd, *J* = 5 Hz, *J* = 5 Hz, 2H, =CH_2_), 5.81–5.86 (m, 1H, CH), 7.34–8.07 (m, 10H, 10ArH).

##### *N*-(5-{[(4,5-diphenyl-4*H*-1,2,4-triazol-3-yl)sulfanyl]methyl}-1,3,4-thiadiazol-2-yl)-*N*-cyclohexylacetamide (**7c**)

Yield: 87.5 %, mp: 193–195 °C (dec.). Analysis for C_25_H_26_N_6_OS_2_ (490.64); calculated: C, 61.20; H, 5.34; N, 17.13; S, 13.07; found: C, 61.22; H, 5.32; N, 17.16; S, 13.05. IR (KBr), *ν* (cm^−1^): 3108 (CH aromatic), 2988, 1487, 755 (CH aliphatic), 1705 (C=O), 1603 (C=N), 1506 (C–N), 674 (C–S). ^1^H NMR (DMSO-*d*_6_) *δ* (ppm): 1.36–1.84 (m, 10H, 5CH_2_ cyclohexane), 2.14 (s, 3H, CH_3_), 3.64 (m, 1H, CH cyclohexane), 4.26 (s, 2H, CH_2_), 7.33–8.05 (m, 10H, 10ArH).

##### *N*-(5-{[(4,5-diphenyl-4*H*-1,2,4-triazol-3-yl)sulfanyl]methyl}-1,3,4-thiadiazol-2-yl)-*N*-phenylacetamide (**7d**)

Yield: 67.7 %, mp: 209–211 °C (dec.). Analysis for C_25_H_20_N_6_OS_2_ (484.59); calculated: C, 61.96; H, 4.16; N, 17.34; S, 13.23; found: C, 61.95; H, 4.08; N, 17.31; S, 13.26. IR (KBr), *ν* (cm^−1^): 3098 (CH aromatic), 2978 (CH aliphatic), 1699 (C=O), 1602 (C=N), 1509 (C–N), 694 (C–S). ^1^H NMR (DMSO-*d*_6_) *δ* (ppm): 2.12 (s, 3H, CH_3_), 4.22 (s, 2H, CH_2_), 7.16–7.92 (m, 15H, 15ArH).

##### *N*-(5-{[(4,5-diphenyl-4*H*-1,2,4-triazol-3-yl)sulfanyl]methyl}-1,3,4-thiadiazol-2-yl)-*N*-(4-bromophenyl)acetamide (**7e**)

Yield: 84.6 %, mp: 222–224 °C (dec.). Analysis for C_25_H_19_BrN_6_OS_2_ (563.49); calculated: C, 53.29; H, 3.40; N, 14.91; S, 11.38; Br, 14.18; found: C, 53.33; H, 3.38; N, 14.95; S, 11.36. IR (KBr), *ν* (cm^−1^): 3123 (CH aromatic), 2974, 1467 (CH aliphatic), 1712 (C=O), 1621 (C=N), 1509 (C–N), 684 (C–S). ^1^H NMR (DMSO-*d*_6_) *δ* (ppm): 2.15 (s, 3H, CH_3_), 4.25 (s, 2H, CH_2_), 7.27–7.94 (m, 14H, 14ArH).

##### *N*-(5-{[(4,5-diphenyl-4*H*-1,2,4-triazol-3-yl)sulfanyl]methyl}-1,3,4-thiadiazol-2-yl)-*N*-(4-chlorophenyl)acetamide (**7f**)

Yield: 59.8 %, mp: 229–231 °C (dec.). Analysis for C_25_H_19_ClN_6_OS_2_ (519.04); calculated: C, 57.85; H, 3.69; N, 16.19; S, 12.36; Cl, 6.83; found: C, 57.81; H, 3.65; N, 16.22; S, 12.37. IR (KBr), *ν* (cm^−1^): 3090 (CH aromatic), 2980, 1451 (CH aliphatic), 1695 (C=O), 1601 (C=N), 1521 (C–N), 689 (C–S). ^1^H NMR (DMSO-*d*_6_) *δ* (ppm): 2.15 (s, 3H, CH_3_), 4.24 (s, 2H, CH_2_), 7.26–7.91 (m, 14H, 14ArH).

##### *N*-(5-{[(4,5-diphenyl-4*H*-1,2,4-triazol-3-yl)sulfanyl]methyl}-1,3,4-thiadiazol-2-yl)-*N*-(4-methoxyphenyl)acetamide (**7g**)

Yield: 62.8 %, mp: 174–176 °C (dec.). Analysis for C_26_H_22_N_6_O_2_S_2_ (514.62); calculated: C, 60.68; H, 4.31; N, 16.33; S, 12.46; found: C, 60.64; H, 4.29; N, 16.37; S, 12.45. IR (KBr), *ν* (cm^−1^): 3067 (CH aromatic), 2987, 1452 (CH aliphatic), 1710 (C=O), 1611 (C=N), 1508 (C–N), 679 (C–S). ^1^H NMR (DMSO-*d*_6_) *δ* (ppm): 2.09 (s, 3H, CH_3_), 3.78 (s, 3H, CH_3_), 3.87 (s, 2H, CH_2_), 7.09–8.50 (m, 14H, 14ArH).

##### *N*-(5-{[(4,5-diphenyl-4*H*-1,2,4-triazol-3-yl)sulfanyl]methyl}-1,3,4-thiadiazol-2-yl)-*N*-benzylacetamide (**7h**)

Yield: 73.4 %, mp: 156–158 °C (dec.). Analysis for C_26_H_22_N_6_OS_2_ (498.62); calculated: C, 62.63; H, 4.45; N, 16.85; S, 12.86; found: C, 62.67; H, 4.48; N, 16.81; S, 12.84. IR (KBr), *ν* (cm^−1^): 3076 (CH aromatic), 2965, 1468 (CH aliphatic), 1713 (C=O), 1614 (C=N), 1523 (C–N), 695 (C–S). ^1^H NMR (DMSO-*d*_6_) *δ* (ppm): 2.06 (s, 3H, CH_3_), 4.26 (s, 2H, CH_2_), 4.75 (s, 2H, CH_2_), 7.19–8.36 (m, 15H, 15ArH).

##### *N*-(5-{[(4,5-diphenyl-4*H*-1,2,4-triazol-3-yl)sulfanyl]methyl}-1,3,4-thiadiazol-2-yl)-*N*-(4-methoxybenzyl)acetamide (**7i**)

Yield: 69.4 %, mp: 201–203 °C (dec.). Analysis for C_27_H_24_N_6_O_2_S_2_ (528.65); calculated: C, 61.34; H, 4.58; N, 15.90; S, 12.13; found: C, 61.37; H, 4.59; N, 15.89; S, 12.16. IR (KBr), *ν* (cm^−1^): 3103 (CH aromatic), 2967, 1461, 756 (CH aliphatic), 1704 (C=O), 1607 (C=N), 1514 (C–N), 697 (C–S). ^1^H NMR (DMSO-*d*_6_) *δ* (ppm): 2.16 (s, 3H, CH_3_), 3.77 (s, 3H, CH_3_), 4.24 (s, 2H, CH_2_), 4.31 (s, 2H, CH_2_), 6.88–7.71 (m, 14H, 14ArH).

##### [(4,5-Diphenyl-4*H*-1,2,4-triazol-3-yl)sulfanyl] acetic acid (**8**)

Compound **8** was obtained using the same method as described earlier for derivatives **5a**–**i**. That is, a mixture of thiosemicarbazide **4j** (10 mmol) and 20 mL of 2 % aqueous solution of sodium hydroxide was refluxed for 2 h. Then, the solution was neutralized with diluted hydrochloric acid and the formed precipitate was filtered and crystallized from ethanol.

Yield: 70.3 %, mp: 248–249 °C (dec.). Analysis for C_16_H_13_N_3_O_2_S (311.36); calculated: C, 61.72; H, 4.21; N, 13.49; S, 10.30; found: C, 61.59; H, 4.19; N, 13.54; S, 10.28. IR (KBr), *ν* (cm^−1^): 3079 (CH aromatic), 3045 (OH), 2982 (CH aliphatic), 1702 (C=O), 1599 (C=N), 688 (C–S). ^1^H NMR (DMSO-*d*_6_) *δ* (ppm): 4.04 (s, 2H, CH_2_), 7.28–7.61 (m, 10H, 10ArH), 12.97 (s, 1H, OH).

##### 4-Carboxymethyl-5-{[(4,5-diphenyl-4*H*-1,2,4-triazol-3-yl)sulfanyl]methyl}-4*H*-1,2,4-triazole-3(2*H*)-thione (**9**)

Compound **9** was obtained using the same method as described earlier for derivatives **5a**–**i**. That is, a mixture of thiosemicarbazide **4k** (10 mmol) and 20 mL of 2 % aqueous solution of sodium hydroxide was refluxed for 2 h. Then, the solution was neutralized with diluted hydrochloric acid and the formed precipitate was filtered and crystallized from ethanol.

Yield: 97.2 %, mp: 157–159 °C (dec.). Analysis for C_19_H_16_N_6_O_2_S_2_ (424.50); calculated: C, 53.76; H, 3.80; N, 19.80; S, 15.11; found: C, 53.88; H, 3.81; N, 19.74; S, 15.47. IR (KBr), *ν* (cm^−1^): 3228 (NH), 3095 (OH), 3062 (CH aromatic), 2991 (CH aliphatic), 1713 (C=O), 1605 (C=N), 1504 (C–N), 1343 (C=S), 681 (C–S). ^1^H NMR (DMSO-*d*_6_) *δ* (ppm): 4.42 (s, 2H, CH_2_), 4.78 (s, 2H, CH_2_), 7.27–7.56 (m, 10H, 10ArH), 13.80 (s, 1H, OH), 14.13 (brs, 1H, NH).

##### 5-{[(4,5-Diphenyl-4*H*-1,2,4-triazol-3-yl)sulfanyl]methyl}-2,5-dihydro-4*H*-1,2,4-triazole-3(2*H*)-thione (**10**)

Compound **10** was obtained using the same method as described earlier for derivatives **5a**–**i**. That is, a mixture of thiosemicarbazide **4l** (10 mmol) and 20 mL of 2 % aqueous solution of sodium hydroxide was refluxed for 2 h. Then, the solution was neutralized with diluted hydrochloric acid and the formed precipitate was filtered and crystallized from ethanol.

Yield: 78.9 %, mp: 210–212 °C (dec.). Analysis for C_17_H_14_N_6_S_2_ (366.46); calculated: C, 55.72; H, 3.85; N, 22.93; S, 17.50; found: C, 55.58; H, 3.83; N, 23.01; S, 17.46. IR (KBr), *ν* (cm^−1^): 3256 (NH), 3079 (CH aromatic), 2956, 1461 (CH aliphatic), 1603 (C=N), 1510 (C–N), 1329 (C=S), 695 (C–S). ^1^H NMR (DMSO-*d*_6_) *δ* (ppm): 4.04 (s, 2H, CH_2_), 7.29–7.92 (m, 10H, 10ArH), 13.33 (s, 1H, NH), 14.15 (brs, 1H, NH).

##### [3-{[(4,5-Diphenyl-4*H*-1,2,4-triazol-3-yl)sulfanyl]methyl}-1-(pyrrolidin-1-ylmethyl)-5-thioxo-1,5-dihydro-4*H*-1,2,4-triazol-4-yl]acetic acid (**11**)

To a solution of 10 mmol of compound **9** in ethanol, pyrrolidine (10 mmol) and formaldehyde (0.2 mL) were added. The mixture was stirred for 2 h at room temperature. After that, distilled water was added and the precipitate that formed was filtered, washed with distilled water, and crystallized from ethanol.

Yield: 66.8 %, mp: 173–175 °C (dec.). Analysis for C_24_H_25_N_7_O_2_S_2_ (507.63); calculated: C, 56.78; H, 4.96; N, 19.31; S, 12.63; found: C, 56.80; H, 4.97; N, 19.34; S, 12.66. IR (KBr), *ν* (cm^−1^): 3100 (OH), 3069 (CH aromatic), 2962 (CH aliphatic), 1715 (C=O), 1611 (C=N), 1514 (C–N), 1367 (C=S), 692 (C–S). ^1^H NMR (DMSO-*d*_6_) *δ* (ppm): 1.66–1.72 (m, 4H, 2CH_2_), 2.29 (t, *J* = 5 Hz, 2H, CH_2_), 2.68 (t, *J* = 5 Hz, 2H, CH_2_), 4.27 (s, 2H, CH_2_), 4.58 (s, 2H, CH_2_), 4.69 (s, 2H, CH_2_), 7.47–8.08 (m, 10H, 10ArH), 13.68 (s, 1H, OH).

##### 5-{[(4,5-Diphenyl-4*H*-1,2,4-triazol-3-yl)sulfanyl]methyl}-2-(pyrrolidin-1-ylmethyl)-2,4-dihyro-3*H*-1,2,4-triazole-3-thione (**12**)

To a solution of 10 mmol of compound **10** in ethanol, pyrrolidine (10 mmol) and formaldehyde (0.2 mL) were added. The mixture was stirred for 2 h at room temperature. After that, distilled water was added and the precipitate that formed was filtered, washed with distilled water, and crystallized from ethanol.

Yield: 74.8 %, mp: 224–226 °C (dec.). Analysis for C_22_H_23_N_7_S_2_ (449.59); calculated: C, 58.77; H, 5.16; N, 21.81; S, 14.26; found: C, 58.79; H, 5.14; N, 21.83; S, 12.24. IR (KBr), *ν* (cm^−1^): 3290 (NH), 3098 (CH aromatic), 2978, 1482 (CH aliphatic), 1623 (C=N), 1522 (C–N), 1341 (C=S), 685 (C–S). ^1^H NMR (DMSO-*d*_6_) *δ* (ppm): 1.67–1.73 (m, 4H, 2CH_2_), 2.32 (t, *J* = 5 Hz, 2H, CH_2_), 2.77 (t, *J* = 5 Hz, 2H, CH_2_), 4.05 (s, 2H, CH_2_), 4.68 (s, 2H, CH_2_), 7.36–8.35 (m, 10H, 10ArH), 14.68 (brs, 1H, NH).

### Microbiology

#### Materials and methods

All synthesized compounds were preliminarily tested for their in vitro antibacterial activity against Gram-positive and -negative reference bacterial strains and next by the broth microdilution method against the selected bacterial strains.

Panel reference strains of aerobic bacteria from the American Type Culture Collection, including six Gram-positive bacteria, *S. aureus* ATCC 25923, *S. aureus* ATCC 6538, *S. epidermidis* ATCC 12228, *B. subtilis* ATCC 6633, *B. cereus* ATCC 10876, *M. luteus* ATCC 10240, and four Gram-negative bacteria, *Escherichia coli* ATCC 25922, *Klebsiella pneumoniae* ATCC 13883, *Proteus mirabilis* ATCC 12453, *Pseudomonas aeruginosa* ATCC 9027, were used. Microbial suspensions with an optical density of 0.5 McFarland standard 150 × 10^6^ CFU/mL (CFUs—colony forming units) were prepared in sterile 0.85 % NaCl. All stock solutions of the tested compounds were prepared in DMSO. The medium with DMSO at the final concentration and without the tested compounds served as the control—no microbial growth inhibition was observed.

Preliminary antimicrobial potency in vitro of the tested compounds was screened using the agar dilution method on the basis of the bacterial growth inhibition on the Mueller–Hinton agar containing the compounds at a concentration of 1,000 μg/mL. The plates were poured on the day of testing. 10 μL of each bacterial suspension was put onto the prepared solid media. The plates were incubated at 37 °C for 18 h (Bourgeois *et al*., 2007).

The antibacterial activity in vitro of the potentially active compounds was determined by the broth microdilution method on the basis of MIC, usually defined as the lowest concentration of the compound at which there was no visible growth of microorganisms (White *et al*., 2002). Determination of the MIC value was achieved by the broth microdilution method according to a CLSI (Clinical and Laboratory Standards Institute) recommendation with some modifications (2008). The 96-well microplates were used; 198 μL of Mueller–Hinton broth with a series of twofold dilutions of the tested compound in the range of the final concentrations from 0.24 to 1,000 μg/mL was inoculated with 2 μL of microbial suspension (total volume per each well—200 μL). After incubation (at 35 °C for 18 h), spectrophotometric measurements of optical density (OD_600_) of the bacterial cultures with the tested compounds were performed in order to determine MIC. OD_600_ of bacterial cultures in the medium without the tested compounds was used as a control. The blank control wells with twofold dilution of each of the tested compounds added to the Mueller–Hinton broth without bacterial suspension were incubated under the same conditions. Cefuroxime, belonging to the second generation of cephalosporins, was used as a control antimicrobial agent.
